# A case report of hereditary hemochromatosis caused by mutation of SLC40A1 gene

**DOI:** 10.1097/MD.0000000000017526

**Published:** 2019-11-01

**Authors:** Xin Yin, Yu Zhang, Hui Gao, Qing-long Jin, Xiao-yu Wen

**Affiliations:** a Department of Hepatology, The First Hospital of Jilin University, Changchun; b Department of Digestive disease, Tai’an Municipal Hospital, Tai’an, China.

**Keywords:** case report, hereditary hemochromatosis, iron metabolism, mutation, phlebotomy

## Abstract

**Rationale:**

Hereditary hemochromatosis (HH) is a frequent autosomal recessive disease. The pathogenesis of disease is excessive intestinal absorption of dietary iron, resulting in pathologically high iron storage in tissues and organs. As a systemic disease, it has several manifestations including cirrhosis, diabetes mellitus, cardiomyopathy, joint disease. However, a proportion of patients are asymptomatic.

**Patient concerns:**

A 34-year-old man who had abnormal liver function for 9 months without specific symptoms. He underwent various tests, including liver biopsy and genetic testing, which eventually ruled out common liver diseases and identified iron metabolic abnormalities. In addition, we confirmed the pathogenic genes by sequencing the genes of him and his families.

**Diagnosis:**

Combined with the symptoms, auxiliary examinations and sequencing results, the patient was diagnosed as HH.

**Interventions:**

The patient was given a low iron diet and phlebotomy therapy interval 2 weeks until the ferritin is <100 mg/L.

**Outcomes:**

The patient’ condition is stable during the follow-up period.

**Lessons:**

When clinicians are confronted with unexplained liver dysfunction, the possibility of the HH should be considered. Liver biopsy and gene sequencing are helpful in diagnosis. Phlebotomy treatment is the most economical and practical treatment for HH at present, but it should vary from person to person.

## 1 Introduction

Hereditary hemochromatosis (HH) is a frequent autosomal recessive disease. The pathogenesis of disease is excessive intestinal absorption of dietary iron, resulting in pathologically high iron storage in tissues and organs. As a systemic disease, it has several manifestations including cirrhosis, diabetes mellitus, cardiomyopathy, joint disease.^[1,2]^ However, a proportion of patients are asymptomatic. There are 4 main classifications of HH, as well as 5 subtypes. In Caucasians, mutations in the HFE-gene are responsible for most cases of HH (type 1). Non-HFE-hemochromatosis is less frequent and consists of hepcidin deficient hemochromatosis including hemojuvelin (HJV type 2A) and hepcidin (HAMP type 2B) and TRF2-related hemochromatosis (type 3).^[2,3]^ The others comprise ferroportin disease (type 4A) and atypical ferroportin disease (type 4B).^[2,3]^ V162del has been reported in non-C282Y hemochromatosis. Here, we report an identified V162del mutation of SLC40A1 in a Chinese-family. This report is the only family report on SLC40A1 caused by V162del in China. Although the gene mutation was mentioned in Zhang Wei data, there was no family report.^[4]^ Our case was helpful for diagnosis and treatment on asymptomatic HH patients.

## 2 Case presentation

A 34-year-old man was admitted to our hospital on August 2014 due to occasional discomfort in the liver area for 9 months. The patient felt fatigue occasionally and had no history of joints pain. The patient denied history of hypertension, coronary heart disease, diabetes, viral hepatitis and tuberculosis, and also denied history of surgery, trauma, blood transfusion, and food or drug allergy. He has smoking history for 7 years (about 7 cigarettes per day), and occasionally drank in recent 5 years (one time per week, equivalent alcohol intake <60 g per time). Nine months before being admitted, he had not received any additional treatment except for taking hepatoprotective drugs.

There was no abnormality in physical examination.

In the laboratory tests, liver function showed that aspartate aminotransferase was 48.5 U/L (reference range: 15–46 U/L) and alanine aminotransferase was 73.1 U/L (reference range: 0–40 U/L). The iron metabolism showed that the serum iron was 23.4 μmol/L (reference range: 10.6–36.6 μmol/L), total iron binding capacity was 47.2 μmol/L (reference range: 50–70 μmmol/L), ferritin was 12,405.0 μg/L (reference range: 20–200 μg/L), and transferrin saturation was 50% (reference range: 20–50%). No abnormal findings in the tests of blood and coagulation routine, urine and stool routine; no abnormal findings in the tests of kidney function, electrolyte, blood lipid and glycosylated hemoglobin; no abnormal findings in hepatitis B markers, hepatitis C antibody as well as alpha fetoprotein (AFP); antinuclear antibody (ANA), autoimmune liver disease-related antibodies, and immunoglobulin were normal. ECG was normal. Echocardiography showed mild tricuspid regurgitation.

**Figure 1:**
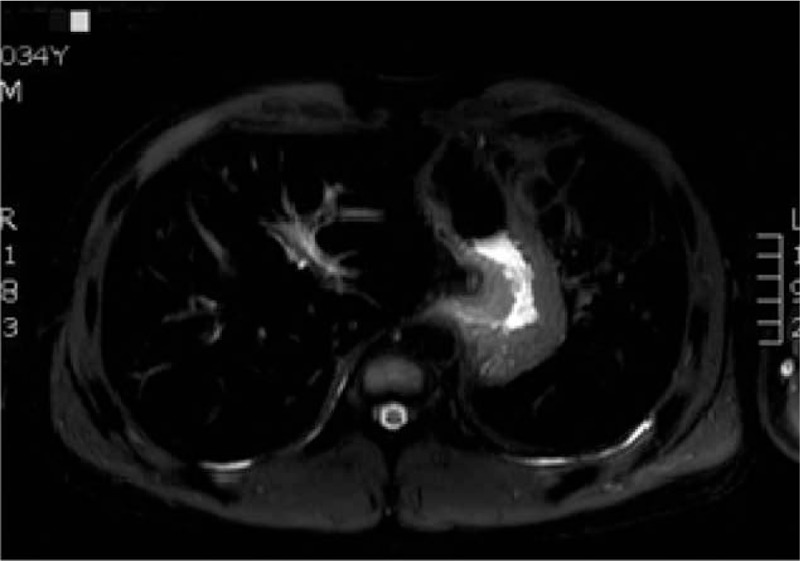
The signal of liver reduced on T2W1, and represented as a “black liver” on MRI scan. MRI = magnetic resonance imaging.

**Figure 2:**
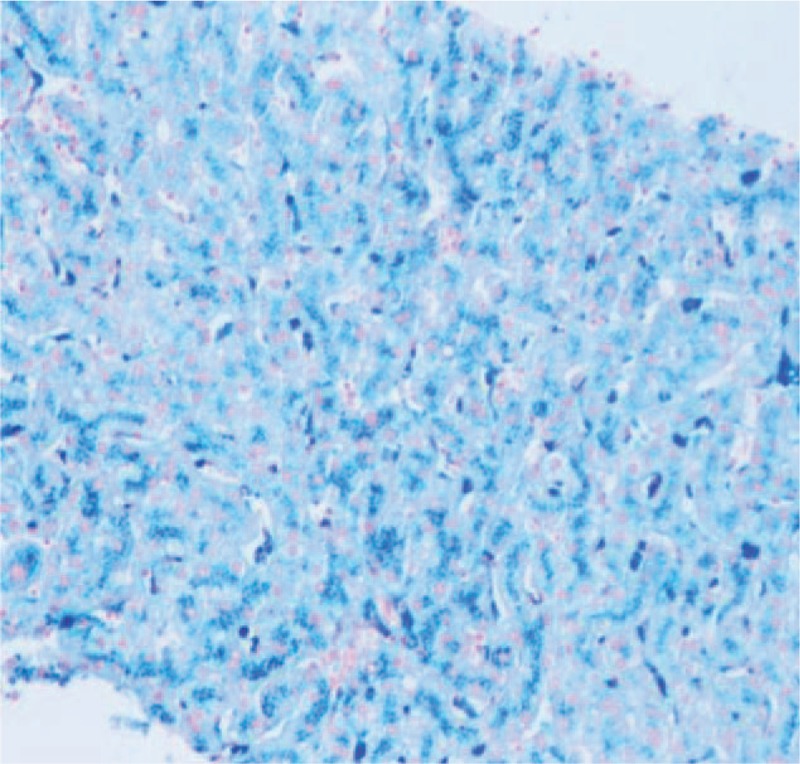
Iron staining of liver tissue (Hematoxylin and eosin staining [HE] ×200). The iron particles were showed as blue color and were mainly deposited in the cytoplasm of liver cells.

**Figure 3:**
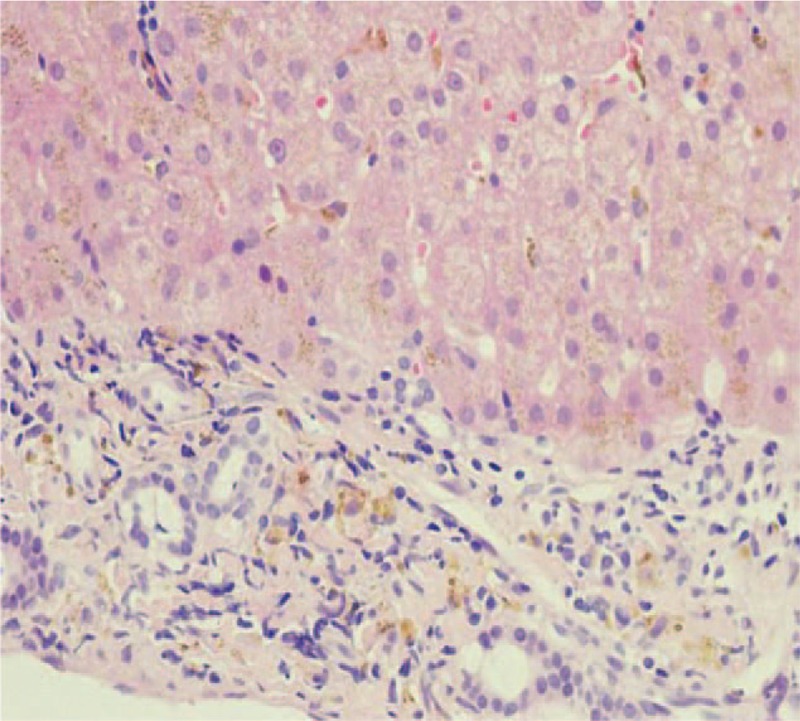
HE showed pigmentary particles deposition in the hepatocyte cytoplasm with refractivity (HE ×400). HE = Hematoxylin and eosin staining.

**Figure 4:**
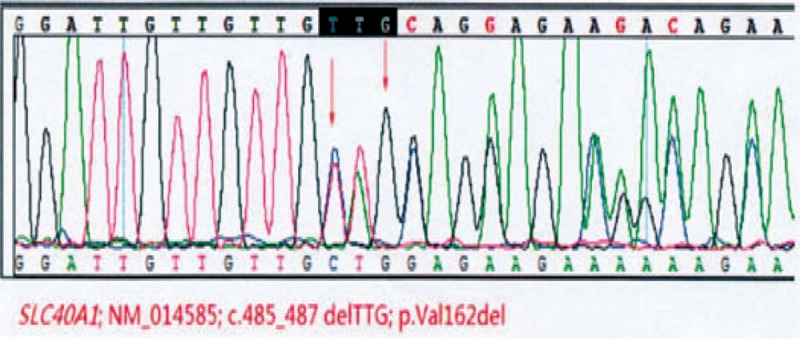
Sequencing result of SLC40A1 deletion mutation in the patient.

**Figure 5:**
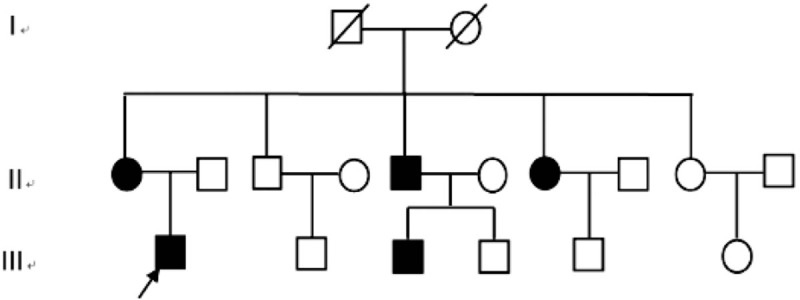
Family pedigree of the mutation in V162del. Family members with V162del are shown as black symbols (arrow = proband). II1: serum ferritin: 9766 μg/L. II5: serum ferritin: 3285 μg/L. II7: serum ferritin: 1933 μg/L. III3: serum ferritin: 2993 μg/L.

Contrast-enhanced magnetic resonance imaging (MRI) of the liver and spleen showed enlarged spleen and extensive and uniform decrease of the signal in liver and spleen (Fig. 1). Liver biopsy showed phagocytic Kupffer cell infiltration, expanded portal area, fibrous tissue proliferation, and a few of inflammatory cells infiltration. Iron staining was positive and copper staining was negative. The pathologic diagnosis was hereditary hemosiderosis (Figs. 2 and 3). Sequencing test was performed on the pathogenic genes in the online Mendelian Inheritance in Man (OMIM) database including HFE, HAMP, HJV, TFR2, and SLC40A1 gene. Gene mutation was not found in HFE, HAMP, HJV, and TFR2. However, it was found that the TTG at position 485 to 487 of SLC40A1 gene was deleted, resulting in the deletion of the valine 162 of encoded ferroportin1 protein. The mutation of the gene shows autosomal dominant inheritance. The patient was heterozygote for the mutation (Fig. 4). Genetic test was further performed on his relatives. It was found his mother, 1 of the 2 aunts, and 1 of the 2 uncles also carried heterozygous mutation of Val162del of SLC40A1 gene (Fig. 5).

The patient was given a low iron diet and phlebotomy interval 2 weeks until the ferritin 100 μg/L. At present, phlebotomy is performed every 3 to 6 months according to the results of the patient's examination. There were no significant changes in blood routine tests. With those treatments, he was asymptomatic with 5 years follow-up.

This case report was approved by the ethics committee of the First Hospital of Jilin University, Changchun, China, and the informed consent form was signed by patient.

## 3 Discussion

HH is an inherited disorder of iron metabolism. It is among the most common autosomal recessive conditions of Caucasian populations.^[5,6]^ The genetic bases for hemochromatosis can be divided principally into HFE gene mutations and non-HFE mutations.^[7,8]^ Hemochromatosis is then further subdivided into 4 overall types. Hepcidin deficiency is the common denominator and is responsible for organ iron excess through increased cellular iron entry. Types I–III are linked to altered or reduced expression of hepcidin,^[7,9,10]^ whereas type IV results from ferroportin mutations.^[8,10]^

Ferroportin is the product of SLC40A1 gene. Ferroportin express in tissue macrophages and at the basolateral side of duodenal enterocytes and placental cells and it is the only known iron exporter in vertebrate cells.^[11]^ SLC40A1 gene mutation results in changes in the structure or function of transferrin, which cause iron deposition in multiple organs. Impaired liver function occurs at an early stage of HH. Timely detection of HH can prevent progress to cirrhosis or hepatocellular carcinoma. The diagnosis of HH is mainly based on laboratory and imaging examination. In clinical practice, the most useful biomarker for estimating systemic iron storage is serum ferritin. It is necessary to consider the possibility of HH when ferritin is elevated (≥300 μg/L in men and ≥200 μg/L in women), excluding inflammation, metabolic syndrome, alcoholism, and so on. At the same time, iron deposits in organs can be determined by MRI. Liver biopsy can further clarify the extent of liver lesions, but it is no longer necessary for diagnosis. Phlebotomy is the standard treatment for HH. The target value of the serum ferritin level is between 50 and 100 μg/L.^[12]^ The time interval of phlebotomy was adjusted according to patients’ tolerance, hemoglobin level, and ferritin level. If the patient cannot tolerate phlebotomy therapy, iron chelation therapy may be considered. Erythrocytapheresis could be useful for the treatment of HH, but it is rarely used in clinical practice. Hepcidin replacement therapy could provide an etiologic cure; thus, the interest on the development of hepcidin therapeutics is growing.^[13]^

In OMIM database, HH was classified into 4 types based on different gene mutations. Mutations in HFE, HJV, HAMP, TFR2, and SLC40A1 have been linked to the various types of hemochromatosis.^[8–10]^ HFE mutation associated Type 1 is more common in European and American populations that are the classic type of hemochromatosis. Types 2, 3, and 4 are collectively referred to non-HFE-associated hemochromatosis and are the major HH types in Asia.^[3]^ Type 2 is further classified into type 2A HJV (HFE2 gene) and type 2B type (HAMP). Type 3 is associated with TfR2 genes, and type 4 is associated with SLC40A1 gene. Different from other 3 types, type 4 displays an autosomal dominant inheritance pattern. The mutated gene is located in 2q32 and encodes ferroportin 1, and missense mutations of this gene were firstly reported by Dutch and Italian scientists in 2001.^[14,15]^ V162del is the most common mutation site,^[8–10]^ which may also be most common in non-C282Y hemochromatosis. To date, 51 SLC40A1 mutations have been identified.^[14]^ SCL40A1 is commonly seen in Europe and the United States, reports in Europe are in Italy, Spain, and France.^[16–21]^ However, the mutation of this gene has rarely been reported in Asia, mainly in Japan, China, and India.^[4,7,22,23,24]^ V162del mutation can lead to the increase of ferritin in the early stage of HH. Liver biopsy indicates iron deposition in Kupffer cells which may reduce the damage of iron on other cells. Patients generally well tolerate to phlebotomy without anemia.^[9]^

With the increase of the reports of non-HFE hereditary hemochromatosis, more and more patients with type 4 which is associated with V162del mutation of SLC40A1 gene were diagnosed. For patients with long-term abnormalities in liver function, in addition to the common causes, genetic liver disease should be further screened. For suspected patients, liver biopsy should be given as soon as possible to confirm liver histological changes, and genetic test should be conducted to clear types of gene mutations as well as further pedigree analysis. Based on sequencing results, asymptomatic patients can be intervened early so that patient prognosis and quality of life can be improved.

## Acknowledgments

The authors are grateful to the patient, who gave his informed consent for publication.

## Author contributions

**Funding acquisition:** Xiaoyu Wen.

**Investigation:** Yu Zhang.

**Resources:** Hui Gao, Qing-long Jin.

**Writing – original draft:** Xin Yin.

**Writing – review & editing:** Xiaoyu Wen.
